# Supplementary material for: Causal interplay between lactose intolerance and gut microbiota: a combined bidirectional Mendelian randomization and in vivo validation study
Source: Front Nutr. 2026 Jun 1;13:1803337. doi: 10.3389/fnut.2026.1803337 (PMC13265576; doi:10.3389/fnut.2026.1803337)
Supplement: Supplementary file 1 [file Data_Sheet_1.zip › supplementary materials/Forward/forest plot/ebi-a-GCST90027634.finngen_R12_E4_LACTONAS.pdf]

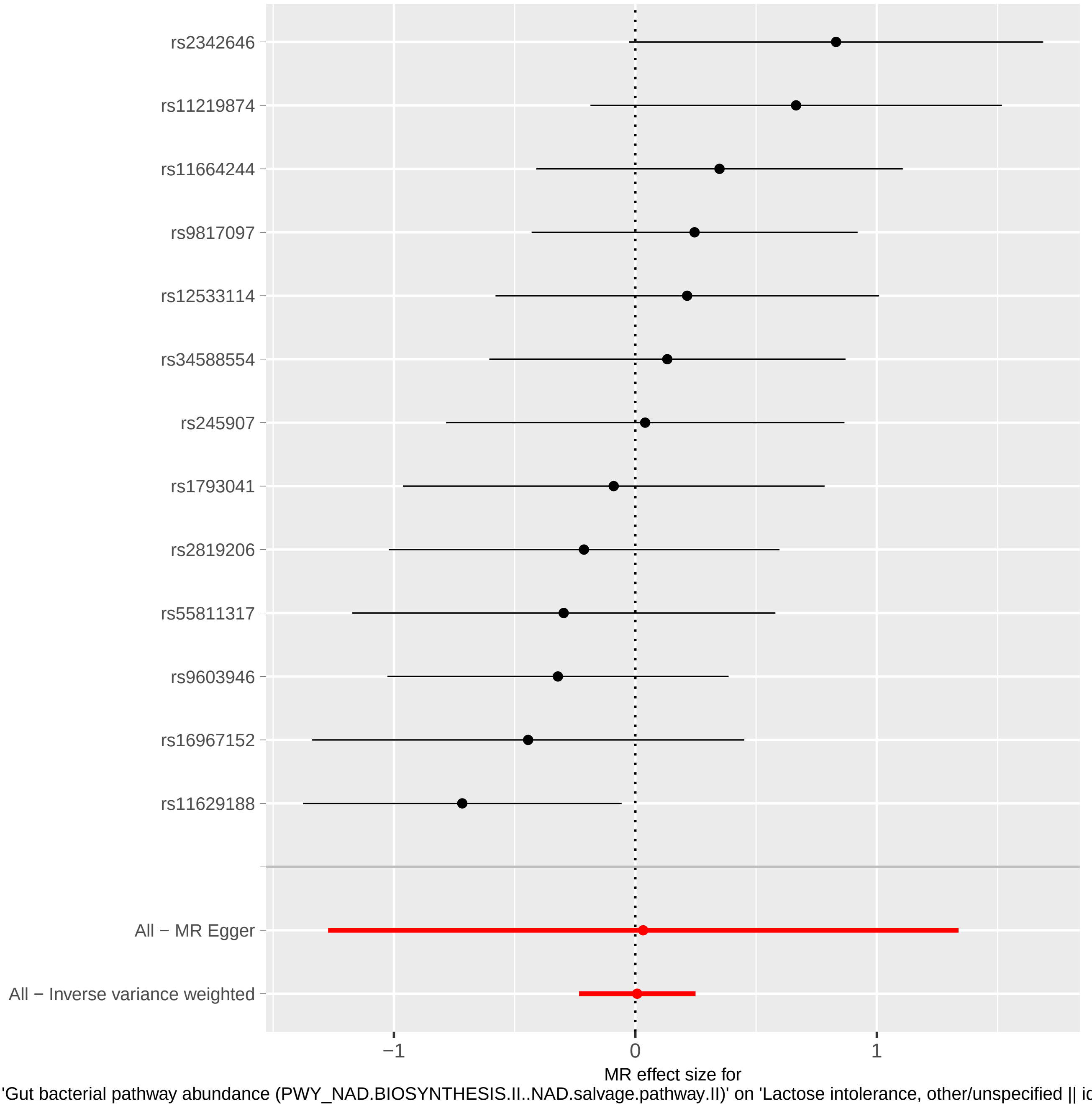
rs2342646
rs11219874
rs11664244
rs9817097
rs12533114
rs34588554
rs245907
rs1793041
rs2819206
rs55811317
rs9603946
rs16967152
rs11629188
All − MR Egger
All − Inverse variance weighted
−1
0
1
MR effect size for
'Gut bacterial pathway abundance (PWY_NAD.BIOSYNTHESIS.II..NAD.salvage.pathway.II)' on 'Lactose intolerance, other/unspecified || id
